# Middle-aged Lebanese women’s interpretation of sexual difficulties: a qualitative inquiry

**DOI:** 10.1186/s12905-020-01132-0

**Published:** 2021-05-17

**Authors:** Mathilde Azar, Caroline Bradbury-Jones, Thilo Kroll

**Affiliations:** 1 Faculty of Health Sciences – University of Balamand. St George Health Complex, Youssef Sursock Street. P.o. Box. 166378 Ashrafieh, Beirut, 1100-2807 Lebanon; 2 School of Nursing, University of Birmingham, Edgbaston, Birmingham, B15 2TT UK; 3 School of Nursing, Midwifery and Health Systems, University College Dublin, Belfield, Dublin 4, UK

**Keywords:** Women, Sexual difficulties, Qualitative research, Marital relation, Context, Middle-age

## Abstract

**Background:**

The study explores women’s perception and experience of sexual difficulties. The need to address the subject was triggered by the scarcity of research that reflects on women’s subjective views on sexual difficulties. This is particularly crucial for middle-aged women who frequently experience hormonal and psychosocial changes that may affect their sexual life.

**Methods:**

Using in-depth individual and focus groups interviews, 52 Lebanese women aged 40–55 years discussed their thoughts, feelings and behaviours concerning sexual difficulties. Women were recruited purposefully from clinical and non-clinical settings to get maximum sampling variation that provided rich information and deep understanding of the subject. Recordings were transcribed verbatim and analysed about the framework analysis. Many strategies were adopted to ensure rigour.

**Results:**

Women’s narratives led to four themes: women’s inability to communicate sexual desires and concerns; male sexual difficulties; marital conflicts; and sexual difficulties as context-bound. Women’s sexual difficulties are driven by double standards and inhibiting sexual socialisation. Once married, many women had very challenging sexual experiences. They were obliged to silently bear their husbands’ poor sexual performance to protect their masculinity and thus their social image and identity. Women’s narratives also showed that marital conflicts, daily life problems as well as physical and psychological burdens further challenged their sexual wellbeing and contributed to their sexual difficulties.

**Conclusion:**

The study makes a unique contribution to voicing women’s views and concerns as sexuality is insufficiently researched and reported in Lebanon. It emphasises the multidimensional nature of female sexual difficulties, particularly the gender-based norms that inhibit their sexual selves and profoundly affect their sexual wellbeing and capacity to claim their sexual likes and dislikes. Findings have implications on research and practice to help women prevent and overcome their sexual difficulties.

**Supplementary Information:**

The online version contains supplementary material available at 10.1186/s12905-020-01132-0.

## Background

Satisfying sexual experiences throughout the life course and the perception of living a good quality of life and experiencing general wellbeing are associated [1, 2]. However, with increasing age, sexual functioning and satisfaction may also change [3]. For women, the transition to menopause constitutes a significant physical and psychosocial challenge at middle-age. From a biomedical perspective, the literature abounds with epidemiological studies about different forms of female sexual disorders affecting women across cultures and age groups [4–6] in both developed and developing countries [7–9]. Sexual problems increase three folds in the climacteric [10] and are 2.3 times more likely to happen from pre to post-menopause [11]. The prevalence is 41% worldwide among reproductive-age women [McCool et al., 2014]; it rises further among postmenopausal women to reach 68 to 86.5% [12].

A systematic review of 135 quantitative studies from 41 countries identified a multitude of factors that predict female sexual problems, confirming their multidimensional nature [13]. The review revealed some protective factors such as having an intimate relationship and communication, getting married at an older age, being sexually educated and having a favourable body image. Women’s sexual function is also affected by the different standards of sexual conduct that are still pervasive in contemporary societies, including Lebanon. The Global Study of Sexual Attitudes and Behaviors (GSSAB) that surveyed 27,500 men and women in 29 countries showed that among sexually active participants, 43% of men and 49% of women reported experiencing at least one sexual problem during the last 12 months [14]. Women scored less than men in their sexual wellbeing [8, 15]. In the male-centred regimes as defined by Laumann and colleagues [15], the rate of women’s satisfaction with sexual functioning was at 39.7–61.3%, whereas this rate was at 64.4–91.1% in gender-equal regimes.

Studies concerning the effect of hormonal decline and ageing on women’s sexual functioning are controversial. Indeed, some aspects of women’s sexual functioning may decline or worsen with increasing age and reduction of hormonal production [3, 16, 17]. But whether and how these changes affect women depends mainly on the way they are perceived [18]. For instance, it is not certain that menopausal changes inexorably affect sexual desire. With ageing, many women report better sexual functioning [19, 20], an increased frequency of orgasm during intercourse and the number of those who claimed never having had an orgasm decreased [21–23]. A study by Berra and colleagues [24] also revealed that menopausal women (36.2%) are less distressed by sexual dysfunction in comparison with premenopausal women (64.5%).

For some women, a low sexual desire did not cause distress in the menopausal transition and did not threaten their relationship with the partner [25]. Moreover, anxiety during sex declined with ageing [16]. However, other women reported negative impacts of menopause on their interpersonal relationships, marital intimacy, satisfaction and stability that may lead to divorce [25, 26]. A satisfying sexual life, on the other hand, strengthens couples’ marital bonds [27].

Female sexual dysfunction as described by the Diagnostic and Statistical Manual of Mental Disorders [28, 29], has is associated with debate and lack of consensus regarding the conceptualisation of this construct. Additionally, epidemiological studies have faced several methodological limitations [30, 31]. The samples are mostly drawn from clinical settings and do not consider the general population and women of different age groups. The instruments used to identify sexual problems focus predominantly on biomedical aspects with less consideration to women’s subjective view and conceptualisation of these problems.

Since female sexuality is a multifaceted construct, it is important to examine it in the context of women’s lives, their experiences, values and beliefs, and the quality of their relationships in addition to biological, psychosocial and economic factors [25, 26].

Over the past decades, increasing interest has been accorded to qualitative research that represents women’s insights and experiences of sexuality and perception of sexual difficulties. But, few of them have focused on sexual difficulties of the middle-aged women. Studies conducted in the middle-East with different samples of women reported one or more sexual problems with a prevalence rate ranging between 26 and 81.5% [5, 32–36]. A comparative study conducted among a representative sample of women aged 45–55 years from Lebanon, Morocco, Spain and the United States did not depict an association between menopausal status and sexual symptoms. Yet, there is a positive association with age [6]. However, the prevalence of sexual symptoms (59%) was the highest among Lebanese women.

Data generated from these epidemiological studies are indispensable to inform practice and offer appropriate services. In the Middle-East and Lebanon in particular, very little research has been conducted to understand sexual life from the perspective of women. As reported by Maaita and colleagues [36], in Arabic societies, talking about sexual disorders is very sensitive and may lead to inaccurate and non-comprehensive results. In Lebanese culture, sexuality and sexual concerns are still taboo subjects, especially for the middle-aged and older generation. At this age, there is still a tendency to position women as asexual. Sexual difficulties are neither discussed publicly nor privately. Healthcare providers seldom discuss the subject with their patients. Like men, women rarely seek help for sexual difficulties, probably because they are unaware of the nature of their problems or simply feel ashamed. Just like in other parts of the world, Lebanese women view sexuality vital for marital stability [37]. Thus, women face the challenge of navigating the tension between maintaining a satisfying sexual life and staying silent on the issue should sexual difficulties arise.

It may not be surprising that sexuality is under-researched and not visible as a topic in the Lebanese health care context. This paper contributes to opening up the discourse on sexuality and sexual concerns. It aims to explore middle-aged Lebanese women’s perception and experience of sexuality and associated difficulties arise as a result of sexual norms that are exclusively defined by men.

An improved understanding of the sociocultural aspects of these experiences will provide critical information for healthcare professionals to engage with women purposefully about their sexuality and related concerns.

## Methods

### Research design

This paper reports on part of a series of qualitative research studies, including different phases where one phase informed the other and provided a comprehensive understanding of women’s sexuality. This naturalistic and holistic paradigm guided by an interpretive and inductive process, is well suited to explore sexuality, which is a complex, tacit, sensitive and no well-known topic [38]. It allows researchers to gain deep insights into women’s thoughts, feelings, attitudes and experiences concerning sexual difficulties as grounded in the Lebanese context.

### Research setting and participants

The sample was recruited from clinical and non-clinical settings. The clinical settings included mammography units of two university hospitals, one private and one public and three outpatient clinics. The non-clinical settings included two organisations, a journalism agency and a business centre. The variation in the settings was suggested to optimise heterogeneity and enrich data generation [39]. Access to the participants required the approval of the administrators of the chosen settings. The first investigator of the study was involved in the recruitment process. She met the potential participants identified by the administrative personnel of the settings, provided them with information about the study in writing and orally. She answered all their questions and gave them time to make their decision. Those who expressed an interest in participating consented to be interviewed after having read an information leaflet detailing their involvement in the research and the ethical considerations. In line with other researchers’ observations [40, 41], the recruitment process was long and difficult as some women were hesitant to reflect on their sexual life, given the sensitivity of the topic.

Women were selected purposively by level of education and menopausal status, acknowledging their implication in the perception and experience of sexuality [42, 43]. Demographic data included marital status, religion and occupation. Due to the sensitive nature of the topic and associated recruitment challenges snowball sampling was used. This concerned only eight women who were identified in this way without compromising the heterogeneity of the sample [44]. Women who participated in the study were 40–55 years old, Lebanese and spoke Arabic, regardless of their marital status and sexual orientation. The women were also asked their health. Those who declared having acute (sudden and severe health problem) or chronic physical and mental health problems (non-controlled diabetes, hypertension, heart disease, cancer, depression, etc.) at the time of the interviews were excluded. Their health condition might have interfered with the perception and experience of sexuality, adding another layer of complexity to the interpretation of findings [45]. Data saturation was considered to have been reached when no new or conflicting information arose in the interviews [46, 47].

### Data collection

The first author (MA) conducted 37 individual interviews and three focus groups, each one composed of four to six participants. Interviews lasted between 45 and 90 min, and focus groups between 90 and 180 min. The two data collection approaches were used as a triangulation method to enhance data richness and depth of inquiry and provide a more comprehensive understanding of women’s sexual difficulties [48].

A topic guide was developed for the purpose of this study with a series of open-ended questions that allowed the participants to reflect on intimate issues and voice their sexual concerns. It is provided as an Additional file 1. To overcome the potential sensitivity and embarrassment about discussing sexuality, a case description and vignette were used to facilitate the conversation [49]. The participants were asked to comment on a situation that portrays a 49 old woman who was interviewed within a program of women’s health about her sexual health issues. Discussion prompts and questions focused on the disclosure of sexual problems and sexual changes during the menopausal period. The vignette introduced the study and prompted participants to reflect on their own experiences. Learning from using this approach also informed how subsequent interviews were conducted. The first author who is Lebanese and qualified in reproductive health due to her background as a nurse and midwife conducted all interviews. The majority of the interviews were carried out at the researcher’s clinical workplace, private and comfortable environment to allow the participants to express themselves freely [50]. Prompts were used to stimulate women to talk, such as: *What words or phrases come to mind that are synonymous with sexual problems for you? What might be the factors that relate to women’s sexual problems? What would you consider to be problematic for you in sexual life?* A notetaker assisted the researcher in focus group discussions. After each individual or focus group interview, field notes with verbal and non-verbal cues, memos and preliminary interpretations were summarised to guide the subsequent interviews and research questions and to get first insights into data generated. The interviews were audio-recorded and transcribed verbatim.

### Data analysis

Informed by Framework Analysis [51, 52], data were analysed in an iterative, analytical and inductive process of comparing and contrasting propositions and developing patterns and themes. This implied different steps which are familiarisation with data, identification of a thematic frame that served to index and chart all transcripts and allow for organising, classifying, comparing and contrasting the extracted data. Mapping and interpretation are the last steps whereby the themes and subthemes were identified and structured in a meaningful manner supported by relevant information. The first steps of the analysis were done in Arabic, the native language of the participants. At the level of charting, we translated codes and data extracts into English and grouped them under headings and subheadings. The rationale for this was to preserve the cultural meaning conveyed by the language and the implications this might have on the accuracy of findings [53, 54].

### Rigour

Credibility or internal validity of findings was checked through reflexivity that was facilitated by the use of an audit trail to keep methodological and analytic documentation, follow the thought processes, track all the decisions made and show transparency and rigour [52, 53]. Transferability was achieved by the thick description of data that was provided through individual and focus group interviews with different purposeful samples chosen from different settings. Within a comfortable environment and good rapport, women extensively reflected on their thoughts and experiences. This generated detailed descriptions and lengthy transcripts. The three authors worked together to agree the coding and analysis process, thus enhancing transparency and dependability of the process. They separately analysed two transcripts that were translated into English by MA whose native language is Arabic.

Moreover, MA translated codes and coded segments into English. These were sorted by the two other researchers thematically arranged. Throughout the analysis process, the three researchers compared and discussed findings until consensus was achieved. We used various strategies to enhance the confirmability of findings. These included a transparent audit trail and peer debriefings.

## Results

### Sample characteristics

Fifty-two women aged between 40 and 55 years participated in the study. Their mean age was 47.40 years. All were married except for five women: one was single, three widowed and one divorced. Sixteen women had enrolled at university, 14 had secondary education (grades 10–12), 12 had an intermediate education (grades 7–9) and ten had an elementary education (grades 1–6) or less. Twenty-eight were housewives. Twenty-seven were Christians and the other Muslims, representing the two dominant religions in Lebanon. Twenty-five indicated that they had started the perimenopause or menopausal period.

### Themes

One-third of the participants reflected on difficult sexual experiences that made them unhappy and dissatisfied with their sexual lives. Most women did not define their sexual difficulties as physical or affected by menopause or ageing. Women’s sexual difficulties were mainly induced by an inhibiting sexual education and patriarchal marital relationships that favour men’s sexual satisfaction and rights. Relationship problems and the burden of daily life overwhelmed women physically and psychologically and also impacted their sexual lives negatively. Key themes in the onset and chronicity of women’s sexual difficulties included the women’s inability to openly express sexual desires and concerns; male sexual dysfunction; marital conflicts; and time- as well as context-bound sexual problems. Sexual difficulties are explained and supported by the participants’ quotations.

#### Women’s inability to communicate sexual desires and concerns

This subtheme refers to women’s sexual self-awareness and experiences in the absence of sexual education and preparedness.

*One cause of sexual problems is* the *absence of sexual education in Lebanon. This is so wrong. She does not know how to discover her body and how to enjoy with her husband. In our society, the woman is not at all aware of sexual life when she gets married. Her husband will not enjoy too. She takes time to discover her body (Faten, university education, individual interview/II).*

After many years of marriage, many women described their deep embarrassment, guilt feelings and shyness around sex. They talked about sex that had been forced on them, loss of desire and vaginismus. Carmen, a woman who had undergone 7 years of psychotherapy said:

*Here you have the taboos, I mean the interdictions, the no, the shame, and this is not allowed … and until now, many women (single young women) do not know why things are not allowed. The shame and interdictions cause sexual frigidity and lead to problems with the husband after marriage because she is not allowed (to be sexually active) before marriage. Suddenly, she gets married, and everything becomes allowed… Psychologically, you are blocked. How she will do it in 24 h? … that is why there are a lot of sexual difficulties among women… (Carmen, university, II).*

Ibtissam’s husband forced himself on her during their first sexual intercourse. She had been a virgin until that time. Meanwhile, her mother and mother-in-law waited outside. Like Ibtissam, Kamal was a university student when she got married. She lost interest in sexuality after her initial negative experiences with her husband. She unsuccessfully tried to negotiate herself out of the ‘tough duty’ as she called it. Sexual intercourse remained a marital duty that had to be performed at the husband’s request.

*I am the type who had an upbringing (conservative). When I got married, I told him: I will iron, wash and clean for you, but do not approach me. Imagine the extent to which we were shy and did not have this. Later on, he helped me get used to him progressively. These things (sex) are not important to me. I am not interested in this thing… I do not like (sex). I do it unwillingly. I am not happy with this… is the least of my worries. I don’t like it. What is important to me is the way he treats me (Kamal, university, II).*

Many participants talked about the perception that women are always ready for sex, and that female sexual problems could only be the result of an illness.

*The woman is stronger than the man in sex. A man might develop impotence and need to take medication... But I rarely hear of women taking these things. Maybe her hormones are stronger … the woman does not have issues with her sexual life unless she is physically ill. A woman is always ready to respond to a man’s needs (Sara, elementary, II).*

Only one participant, Racha, frankly talked about her pain during sexual intercourse due to vaginal dryness. She was grateful that her husband was patient.

*Now that I have no menses, a quite long time ago, I feel dryness. I mean… it is dry, this is embarrassing. I mean it hurts me, yes I have pain during sex. But my husband is so patient… Thank God… (Racha, secondary, Focus Group/FG).*

Racha’s experience triggered two women of the group to reflect on their husbands’ sexual selfishness.

#### Male sexual difficulties

Many participants associated sexual difficulties with their husband’s problems (e.g. erectile dysfunction, early ejaculation). They also accused their husbands of being selfish. Some women reported being forced to engage in anal sex. They furiously challenged their husbands’ sexual behaviours describing it as unpleasant and illegitimate, banned by the society and religion particularly by Islam. Ten women reported feeling sexually dissatisfied due to their husbands’ erectile and ejaculatory problems. Most husbands did not want to talk about the issue and thus the situation remained unresolved.

Mada, a 46-year menopausal woman with intermediate education who complained of her husband’s erectile dysfunction, said:

*… Because he does not answer me, I say that there is no need to talk… There is no need at all; it makes no difference (whether I tell him or not)… I do not confront him… I feel that he avoids me because he has poor sexual performance… I ask him, but he never answers me. This is frustrating… So I avoid talking with him about these issues (Mada, intermediate, II).*

Mada stated that she and her husband had not had sex for the past year and they did not even talk about it. During the interview, Mada looked frustrated. The need to share her concerns was apparent, considering that her sexual difficulties impacted profoundly on her, physically and psychologically.

Some women interpreted their husbands’ attitudes and behaviours as sexual abuse and neglect. Yet, they felt powerless due to the lack of resources and the cultural norms that do not recognise their sexual rights. Most women chose to keep quiet and accepted their situation as something that could not be changed. Karine, a 43-year-old woman with limited formal education said:

*But what can a person do; in our community, once the woman gets married, it is finished. You get married, and you get children. You have tried to solve your problems, but you failed. What do you do? What is the solution? There are children. You have to sacrifice either your children or yourself. I sacrifice myself (Karine, elementary, II).*

Karine said she put up with her husband’s sexual problems since they got married 21 years ago. Her husband had urged her to keep quiet about the problem, and it took Karine a while to open up about it. This happened only after she had been repeatedly assured that the interviews would be confidential.

Lana experienced similar problems with her husband who was sexually impotent. She expressed her rights for sexual pleasure and criticised the gender-based social norms that overlook women’s sexual needs. She said:

*The man sleeps with his wife and does nothing… You feel that you become used to that… He (her husband) enjoys it, and that is. It does not count if the woman enjoys or not. This is also my right, my right. If the situation was reversed …., I do not think that he would have been patient... He would have blamed me 20 times. He would have cheated on me or married another woman … But when the problem is from the man, you have to keep quiet (Lana, secondary, II).*

Despite her anger, Lana, like most of the women, expressed her readiness to seek help if that were necessary.

*But I am not keen (for sex), it is just for him (her husband); so that he does not say that I neglected him... If he likes it, no, I try; I try to do everything (to please him)… If my husband wants (sex) and I have sexual dysfunction, I treat myself. But if my husband is impotent* ***,*** *why do I have to treat myself?*

Other women spoke about undesirable and painful practices such as forced and anal sex. As articulated by Sara:

*Sexual difficulties? Of course*

*I told you when he goes beyond the limits and wants to have anal sex. These problems happen. I do not accept this because I am not an animal to be treated in this way. If he loves me, he has to take care of me so that I give him this thing from my heart and with love (Sara, elementary, II).*

Sara convinced her husband that anal sex is unhealthy and might cause fatal infectious diseases. Yet, Zeina who faced the same problem was unable to change her husband’s “sexual perversion” Once she became economically independent, she stopped having sex with him. Zeina reported that she had developed psychosomatic problems that required psychotherapy.

*I am a human being, like him, I have my feeling, my, my, my,... [silence], what can I say? Because of that, I am suffering. I can not ventilate; I cannot satisfy my needs. This caused me a lot of pain, everywhere in my body (with tears in her eyes), and anger (Zeina, secondary, FG).*

#### Marital conflict

Nearly all women considered marital relationship problems as the source of sexual difficulties. Mounting day-to-day hassles paired with poor communication among the partners caused or aggravated marital conflicts. Women developed negative feelings towards their husbands, resenting them or becoming indifferent to them. These sentiments inhibited sexual desire and culminated in sexual difficulties between the spouses. Without pleasure, sex is solely a marital duty and the women feel abused. This in turn further fuels resentment towards their partners.

One participant declared that at the beginning of her marital life, sex was pleasurable. Presently and because of marital conflicts, she had feelings of rejection and anger towards her husband. She stopped caring for him and completely lost sexual desire and the ability to orgasm. This was also the case for Sally, who described herself as sexually disinterested and said that she does not love her husband.

*By the way, sorry, I do not have any sexual desire. I do it only at his insistence—no way, impossible. I do not have the desire. There is no way that I feel like it. I do not love him (Sally, university, FG).*

Sally supposed that she would experience sexual desire if she was with another man, implying that sexual difficulties are relational rather than biological. The other women of the group supported Sally’s opinion and gave testimonies of their sexual life to ascertain that *“women could not be driven by instinct”* as they said. Another example was illustrated by Quinana, who declared that the primary purpose of participating in the study was to talk about what she described as a ‘miserable sexual life’. She described herself as an ‘automated sex robot’ for her husband, who she described as rude and abusive. She stated that every time she has sex, she hates herself more and more.

*My husband makes me feel that I am only important for this thing (sex). Because of that, I hate sexual life. I need to feel that I am a woman and then take what you want… I accept everything, poverty, misery, taking care of the house, cooking food every day… but I need him to value my efforts and then you can take from me whatever you want.* With a firm tone, she said*: I give you; you do not take by yourself; I give you… This thing makes me sad… I do not have any rights… I like sex to happen with love… (Quinana, secondary, II).*

When asked about why Quinana continues to have sex with her husband, she replied that she obeys him; otherwise, he violently beats her in front of the children and seriously harms her. Lacking love and respect, she reflected on her situation with apparent bitterness.

Uguette was another victim of her husband’s moral, physical and sexual abuse. She courageously voiced her disappointment in front of women in one of the focus groups:

*When you get married, you say that this is the person that I want to live with... get joy with him, and found a lovely family... You devote yourself, body and soul; then you are confronted by the truth… The man you dreamed about disappointed you; you become disgusted with him... if he sleeps with you, you do not want him. You just want him to finish and leave you alone. This means that you are not happy, and sexual issues are inhibited... It is very important to feel that you are desired to feel that you are a woman (Uguette, secondary, FG).*

Conscious of the violation of her sexual rights, Uguette described herself as a passive recipient who is filled and left like ‘garbage’. Although she was the wage earner of the family, she ‘sacrificed’ herself for her children and refused to leave the house to avoid social stigma.

The women were tired of their husbands’ selfish sexual behaviours. It generated marital conflicts which in turn, intensified their sexual difficulties.

*Sexual difficulties happen if the man is self-centred and wants to enjoy himself without caring about his wife… the man does not control himself… the insistence on his wife to always have sex just for himself… it is very important that the woman enjoys… This is in his nature; he does not care about his wife if she enjoys or not… Another issue relates to man insistence to have sex regularly ignoring her fatigue and concerns... His instincts drive man. In the majority of sexual relationships, women do not enjoy. It is all about the psychological context of the sexual relation (Dalia, university, II).*

In the same line, Tressy said:

*Life is not fair. It is the power of the stronger. My husband is not at all like this. In the end, you are tired, and you lose your interest. You become unhappy with sex with everything. Your life is affected. Sex is not just an act; only a duty?! no… (Tressy, intermediate, 45 years, premenopause, FG).*

#### Sexual difficulties as context and time-bound

In this subtheme, sexual difficulties are framed within the overwhelming challenges the women face in their daily lives as wives, mothers and workers. For instance, Tamara, who is the only breadwinner of the family, explained:

*If I have poor sexual performance, this does not mean that I do not want (to have sex). The problems of life are enormous, and you reach a point where you are tired… Social, financial and family burdens affect it (sexual life) without a doubt and cause sexual difficulties. You need suitable conditions to accept the other… I often experience these situations… (Tamara, university, FG).*

The conversation between two women in a focus groups supports this:

Tressy. *So, at some point, if nothing is happening between a man and a woman [meaning sex], I don’t know if it is caused by poverty or worries … (Tressy, intermediate, FG).*

Sally approved Tressy’s opinion. She said:

*As for me, anything in the society, like what she said, the money, anything may affect me. My husband! No, no way! Nothing can affect him. As for me, if he does not work for 1 day, I am troubled… (Sally, university, FG).*

Sally added:

*If my daughter is sick, I get troubled. Anything can make me nervous. So no way (making sex).*

Tressy continued by saying:

*Sexual life fades between the spouses. There is boredom, quarrel, and sexual life fades with the burden of life.*

Reproductive health burdens like delivery, breastfeeding and infertility can further exacerbate sexual difficulties. Mirvat, who has been facing infertility problems for 7 years, said:

*The feeling that you have put in the extra effort at the expense of your psychological status to stimulate the desire of your husband who himself might arrive from his work hungry and not in the mood because the timing is right. This is one of the worst things that might happen in marital life… At this moment, (she stopped talking and then in a hesitant voice said) I feel destroyed and often (she cries) I have uncontrollable crying when I finish (Mirvat, university, FG).*

Mirvat’s account was quite compelling and made all women in the focus group sympathise with her; they gave her advice to reduce her distress.

Rayan spoke about negative body-image and low self-esteem due to obesity. She viewed herself as unattractive as she did not conform to the female ideal. It negatively affected her sexual desire and ability to satisfy her husband’s sexual needs. This, in turn, made her more frustrated. She stated:

*I am upset because I feel sad; I cannot wear what I want. I do not like to sleep with my husband a lot. I pretend that I am tired. I am intimidated because of my obesity. This bothers me. I have been in this way for 5 to 6 months. I can’t wait anymore. He used to enjoy it, but I didn’t. My husband is still young, only 50 years old (Rayan, elementary, II).*

## Discussion

This study explored the perceptions and experiences of sexual difficulties among middle-aged Lebanese women. Findings revealed that sexual difficulties are rarely the result of simple biological causes. They arise on the basis of a complex and prolonged interplay of socio-cultural, relational and personal factors. The complexity inherent to sexual difficulties challenges a medicalised understanding and lends support to the ‘New View’ model of women’s sexual difficulties [55].

Many women in the study struggled to communicate about sexual desires and difficulties as they either lacked the awareness, vocabulary or confidence to do so. Most felt shy and embarrassed at first but then shared openly their experiences. Many said that they had not been prepared for sexual life. They had not learned about sexual stimulation, arousal and pleasure before they got married. The limited sexual understanding and inhibition had constrained their active sexual engagement with their partner [56]. This in turn had let to misunderstandings in their relationships and paired with a lack of communication to frustrations and sustained unmet sexual needs. Most women were already in their thirties and educated when they got married. However, they were unable to overcome their sexual inhibitions. Negative experiences with the first sexual intercourse affected women’s interest in sexuality [57]. In many cases, the negative stance prevailed over time. A qualitative study by Bellamy and colleagues [58] suggests that many women attribute sexual problems to physiological causes rather than seek a psychosocial explanation. The authors argued that ‘problems arising from psychological difficulties were not seen as real problems and more akin to a good excuse’ [58 , p. 3242].

At this time in their life, many women consider themselves as undesirable or unattractive [26, 59]. They may develop a negative attitude towards sex and could feel ashamed of being sexually active [5]. Adding to that, in patriarchal societies, women cannot talk about their sex life as this is a taboo and private topic [49, 60]. Another assumption could be that women are silent about their sexuality to hide their sexual problems.

Women described how erectile and ejaculatory problems and unpleasant sexual behaviours such as forced anal sex affected their sexual satisfaction. Previous studies reported these findings as significant risk factors of female sexual problems [9] like orgasmic and lubrication difficulties [61] and sexual disinterest and dissatisfaction [62, 63]. The male-centric view dominates women’s sexual life. Typically, sexual fulfilment is genitally focused [56, 64]. Women are socialised to act based on their social gender, perpetuating a perception of sexual life as equivalent to vaginal-penile intercourse. This predominant misconception about normative heterosexuality is a challenge for men and women when they have poor performance. Women’s capacity to negotiate sexual fulfilment away from the genitally focused sexual behaviour and the ‘imperative’ is fundamental.

For women in this study, menopause is associated with an end of sexual activity in the Lebanese context. Sexual concerns or symptoms may be masked by this reductionist sociocultural understanding. Thus, many genuine sexual concerns of women remain unaddressed. Women sexual distress was reinforced by their husbands’ reported resistance to admit the men’s sexual problems and seek help. It might be that men do not want to be seen as weak; yet, women perceive their attitude as an additional evidence of the neglect of their needs. Rather than voicing their concerns, they silently shoulder their husbands’ burden as their lot in life and accord particular attention to their husbands’ ego at the expense of their wellbeing. A paradox surfaces between women’s portrayal as sexually passive and submissive and their responsibility to protect their husbands of social stigma as their sexual performance is equated with their masculinity. Women might also interpret men’s poor sexual performance as their failure questioning their femininity and seducing capacities**.** As also revealed in previous studies, findings suggest that gender imbalance of power reinforces women’s devotion and sacrifice for their partners [42, 49].

Many women perceive the request for anal penetrative sex as inconsistent with socio-culturally accepted and internalised rules and behaviours. Islam considers this a perversion, which is not legal and justifies the divorce. For Muslim women this creates a particular dilemma, as they ought to refuse the request to remain true to the Islamic guidance but would risk anger from their husbands who could reject them. Many women choose passive devotion to their husbands to sustain their family and livelihood [58, 65].

The quality of the relationship is essential to women’s experience of sexual difficulties. Intercourse by itself is not the aim for women. Their perception of the relational context and the quality of the communication with the partner seem to be strongly associated with their sexual interest [57]. Love, romance, and intimacy trigger sexual desire [37, 66]. Marital conflicts exacerbate women’s sexual difficulties. Otherwise, they perceive themselves as sexual objects for their husbands’ pleasure as well as victims of their moral, physical and sexual abuse. Women’s expectations about sex is linked with sexual interest [57]. This confirms that women’s sexuality is not only genitally focused but multidimensional. Basson [67, 68] suggests that sexual life and intimacy are mutually dependent as one affects the other; when sexual activity does not provide women with affection, they are not encouraged to have sex. The claim of some women in this study that sexual life is not worth the price if the marital relationship is not good is supported by other qualitative [58, 69] and quantitative research [70, 71].

The violation of women’s ideals inhibits their feelings, mutes their sexuality and produces sexual problems and dissatisfaction. Appropriate communication between partners has a positive effect on the quality of their relationship. Positive relationship quality, in turn, increases sexual satisfaction.

Women’s sexual difficulties are context and time-bound. Being overwhelmed by daily life events and stressors affects their ability to feel sexual as was also confirmed by Bellamy and colleagues’ study [58]. In a list of pressing needs, they tend to relegate their sexuality to a second plan. According to Maslow’s ‘Hierarchy of Needs’ (1943), sexual fulfilment is difficult to achieve, given other priorities. Another finding that recognises the multidimensional framework of sexual difficulties is the interference of women’s reproductive concerns [breastfeeding and infertility] and psychological problems [low body-image and depressive symptoms] with their sexual interest and pleasure. In a heterosexual relation, concern about body image, weight, attractiveness, physical and psychological conditions and general wellbeing affect women’s sexual functioning and satisfaction [72, 73].

## Conclusion

Findings enrich the social science literature about a sensitive topic that is poorly discussed, particularly among middle-aged women generally and in Lebanon specifically. Women in this study voiced their sexual difficulties from a feminist perspective that considers their views as fundamental. It is important for health professionals to recognise the multidimensional nature of women’s sexual difficulties and develop their knowledge and skills to respond to women’s concerns appropriately. It is erroneous to limit women’s sexual difficulties to a biological cause that could be alleviated by a pharmaceutical drug and neglect all other related factors. A comprehensive sexual education should be offered to all people with different age groups and backgrounds to recognise the cues and importance of sexual life and know how to manage it. It should mainly target the couples given the impact of their interrelation on one another sexual satisfaction. The first sexual experiences tend to shape later experiences, particularly for women [57]. The latter should be mainly targeted to understand and voice their sexual needs and rights, and men to be attentive and responsive to theirs. More studies are needed using qualitative designs to generate further information grounded in women’s context considering different age groups, sexual orientations and marital statuses.

## Supplementary Information

**Additional file 1.** Topic guide of the individual and focus group interviews with women.

## Data Availability

Study participants consented to data sharing only with the research team, and not for additional data use purposes. This is in line with current GDPR legislation. While we acknowledge and appreciate the application and use of FAIR data principles and open data sharing, the sensitive nature of the topic in the Lebanese context and the potential of recognisable and identifiable circumstances may place participants at risk. Thus, we will be unable to share the full dataset as de-identification would be impossible.

## Abbreviations

GSSAB: Global Study of Sexual Attitudes and Behaviors
*II*: *Individual Interview*
*FG*: *Focus Group*
