# Supplementary material for: Middle-aged Lebanese women’s interpretation of sexual difficulties: a qualitative inquiry
Source: BMC Womens Health. 2021 May 17;21:203. doi: 10.1186/s12905-020-01132-0 (PMC8127220; doi:10.1186/s12905-020-01132-0)
Supplement: Supplementary file 1 — Additional file 1. Topic guide of the individual and focus group interviews with women. [file 12905_2020_1132_MOESM1_ESM.docx]

***Topic guide of the individual and focus group interviews with women***

| Participant’s name and pseudonym *………………………………….…………………………...*  *Date ………………………Place of the Interview …………………….…….…………………….*  *Recruitment place ...……………….………………………………………………………………*  *Comments………………………………………………………………………………………….……………………….…………………………………………………………………………...* |
| --- |

**Introduction**

The purpose of this interview is to explore how the middle-aged women understand sexual problems and how their views are shaped. The topic guide comprises a series of open-ended questions about the subject and questions about the sociodemographic profile and menopausal status. You are kindly requested to reflect on your views and experiences concerning the following questions.

**Questions**

How do you describe your sexual life?

| *Probe* | *What are the characteristics of your sexual life? How do you feel about your sexual life? What are the things that you expect to have within sexual life?* |
| --- | --- |

What would you consider to be a sexual problem? Can you explain this? Why do you think this is the case?

| *Probe* | *What words or phrases come to mind that are synonymous with sexual problems for you? What might contribute to women’s sexual problems?* |
| --- | --- |

How do sexual problems affect women’s lives?

| *Probe* | *How personal, marital, family life and other issues might be affected by sexual problems?* |
| --- | --- |

Please describe any sexual problem that you have encountered in your life. How has this affected you?

| *Probe* | *What would you consider to be problematic for you in sexual life? Why this has happened? What are the consequences of this problem on your personal, relational and family life?* |
| --- | --- |

1. Any other comments or something you would like to say?

**Socio-demographic data and menopausal status**

| 1. How old are you? ------------------------------------------------------------------------------------ 2. What is your current marital status? ------------------------------------------------------------- 3. If married, from how long? ------------------------------------------------------------------------ 4. What is the highest education level you have got? -------------------------------------------- 5. In which category do you classify your economic status?   □ Good □ Medium □ Low   1. What is your current employment status? ------------------------------------------------------ 2. What is your religious affiliation? ----------------------------------------------------------------- 3. How do you currently describe your menstrual cycle?    1. □ Regular menses    2. □ Irregular menses have occurred in the past 3 months (3 - 12 months)    3. □ Menses ceased at least 12 months ago |
| --- |
